# Efficacy of Tamsulosin alone versus Tamsulosin Phloroglucinol combination therapy for medical expulsion of lower Ureteral calculi

**DOI:** 10.12669/pjms.342.14134

**Published:** 2018

**Authors:** Muhammad Nadeem Shafique, Mujahid Hussain

**Affiliations:** 1 Dr. Muhammad Nadeem Shafique, Masters in Surgery (MS) Urology, Department of rology, Sialkot Medical College, Sialkot, Pakistan; 2 Dr. Mujahid Hussain, PhD, Department of Biology, FG College, Sialkot Cantt, Pakistan

**Keywords:** Combination therapy, Medical expulsive therapy, Phloroglucinol, Tamsulosin, Ureteral calculi

## Abstract

**Objective:**

To see whether phloroglucinol-added tamsulosin therapy exhibits better efficacy than tamsulosin alone in medical expulsion of lower ureteral stone (LUS).

**Methods:**

Sixty four consecutive adult patients presented in a urological setting at Sialkot, Pakistan between January 2015 and December 2016 with solitary, unilateral 3-8mm sized lower ureteral stone (reported by noncontrast computed tomography of the kidney-ureter-bladder) were documented. Group either study or control was allotted, randomly. Same 0.4 mg tamsulosin, once daily was given to all the participants. However, additional 40 mg phloroglucinol, thrice daily was advised for study group (n = 32). The therapy terminated on confirmation of stone expulsion otherwise continued for 6 weeks. Patients were asked to use 50 mg diclophenac Na on colic episode.

**Results:**

Demographic characteristics revealed 81.2% (n = 52) male patients while age statistics as *M* = 42.3, *SD* = 5.93 (range 32-60) years. The study group showed higher stone expulsion rate (100%) and time to expulsion (*M* = 10.34 days) than control. The values were statistically significant (*p* = .02 and *p* = .0001; χ^2^ test in SPSS). Similarly, combination therapy had advantage on mono therapy for reporting statistically lesser numbers of colic episode (p = .03) and consumption of analgesic (p = .02). A marked difference in rate of adverse effects i.e. 68.8 vs. 90.6% was observed in study and control groups.

**Conclusion:**

Phloroglucinol-added therapy is a better choice for expulsion of LUS than tamsulosin alone with reference to stone expulsion rate and medication time.

## INTRODUCTION

Gradually increasing incidence rate of kidney stone is a significant concern of medical world. Genetics and/or life style e.g. stay in hot humid climate accelerate the urolithiasis – the kidney stone formation. Males are at higher risk of incidence than females; a probable impact of endocrinological differentiation. Similarly, likelihood of its occurrence maximizes at the age around 30 years. Sometimes, it is stuck up in ureter especially distal ureter; hence called as lower ureteral stone (LUS) and causes intense flank pain beside urinary obstruction. Stone removal is the only remedial step. However, unlucky sufferers especially children may face the same situation after few years.^1^ This poses extra financial burden in the family and pressure on public health sector.

A medical practitioner decides type of therapeutic modality for ureteral stone expulsion/removal on clinical manifestations and diagnostic findings. Medical expulsive therapy (MET) is opted for the sufferers agreed to waiting management.^2^ Here, patient is free to move for daily functioning without any hindrance like hospitalization. Higher stone expulsion rate, lower health risks, cost-effectiveness, and a chance to avail minimal invasive treatment (on failure) are some of its salient features. Similarly, taking diclophenac Na – the analgesic on colic episode makes the person tension-free.^3^ So, the MET successfully covers psycho-physiological dimensions of an undergoing beneficiary.

Smooth muscle makes wall of the ureter. Internally, it is lined with alpha-1 adrenergic receptors particularly in lower 1/3^rd^ portion of the ureter also called distal ureter. The receptors detect the stone and stimulate peristalsis for its passage. Otherwise, blockade of receptors (by stone) leads to spasm in stone-surrounding muscle, local edema and inflammation. Colic pain develops when peristalsis attempts to push the stone through the inflamed region. An antagonist of the receptors reverses the mechanism^4^; hence facilitates the stone expulsion. Similarly, analgesic obstructs the transmission of pain stimulus to central nervous system. So, clinicians recommend therapy in the light of stone size as well as controlled symptoms.^5^ Tamsulosin is the choice of physicians for two reasons *viz*. high stone expulsion rate and short expulsion time. Similarly, the success rate of phloroglucinol as an anti-spasmodic drug^6^ is appreciable.

The option of combination therapy minimizes the chance of drug tolerance. Published literature has success stories of tamsulosin or phloroglucinol in combination therapy for expulsion of distal ureteral stone.^7^-^9^ However, there is no evidence of phloroglucinol-added tamsulosin therapy for the same purpose. This is why present work was planned to see whether addition of phloroglucinol with tamsulosin enhances the efficacy for LUS expulsion compared to sole tamsulosin. The findings will motivate scientific community to test-retest the modality before recommendation in countries like Pakistan^10^ where MET is the only readily available therapy for the LUS.

## METHODS

The prospective experimental study was carried out in the Kidney Centre – a urological setting at Sialkot, Pakistan between January, 2015 and December, 2016 after getting clearance from the hospital ethics committee.

Sample size (n) was calculated vide a formula ‘z^2^pq/d^2^’ using p = 18% (derived from secondary data of the centre). Consecutive adult patients with a ureteral 3-8mm sized stone lodged below common iliac vessels as confirmed by computed tomography (noncontrast) were registered provided diclophenac injection relieved pain in 24 hour. However, sufferers with hydronephrosis, renal failure, diabetes, peptic ulcer, user of β-blockers, calcium antagonists, or nitrates (as treatment); pregnant or lactating mothers; who demanded urgent stone removal or refused to give written participation consent were excluded. Random group allotment was ensured to make two equal groups (n = 32) using computer-generated table. Furthermore, baseline information of each recruiter was documented before medication.

Patients in study group were given tamsulosin 0.4 mg once daily with phloroglucinol 40 mg twice daily. However, tamsulosin 0.4 mg once daily was advised for matched control. Drugs were continued until stone removal or for a maximum six weeks as suggested by Kumar and associates.^8^ Need-based use of 50 mg diclophenac Na tablet was recommended on colic pain. Furthermore, patients were educated to use purpose-built mesh net to notice stone expulsion. On reporting of stone expulsion, the patient was evaluated by noncontrast computed tomography of the KUB (kidney-ureter-bladder) along with physical examination, serum creatinine, and urine culture. Failure in stone expulsion (after 6 weeks) was dealt with extracorporeal shock lithotripsy.

Continuous variables were subjected to analysis for mean+/-standard deviations and compared by Mann Whitney U test for non normal distributions after normality test. Discrete variables were processed for rates/frequencies and evaluated through chi-squared test for association and risk estimates. Data were entered in worksheet of SPSS ver. 16.0 (SPSS Inc., Chicago, IL, USA) one by one before analyses and interpretations. In both the tests, a p-value (<.05) was regarded as statistically significant.

## RESULTS

**Fig.1:**
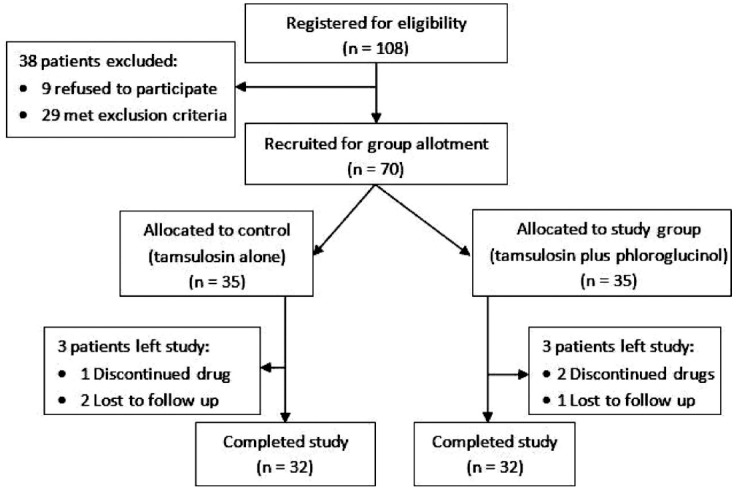
Flow diagram of subject sampling.

Sixty four (91.4% of total 70) patients completed the therapeutic session for expulsion of lower ureteral stone. The remaining were dropped out on drug(s) discontinuation or lose of follow up as shown in flow diagram of subject sampling (Fig.1).

Table-Ia indicates statistics of baseline information of the participants. Statistically insignificant difference in mean age of participants of study group (*M* = 43.84, *SD* = 6.7; range 34-60) years and control was observed using Mann Whitney U test for non normal distributions (*p* = 0.29). Similarly, overall male population 52 (81.3%) dominated. In 62.5% (n = 36) patients, the single stone was diagnosed in left ureter by noncontrast computed tomography (CT) of the kidney-ureter-bladder. Similarly, the size of stone in study group (*M* = 5.47mm in the biggest dimension i.e. axial plane) and control (*M* = 4.50 mm) showed significant difference (*p* = .0001).

**Table-Ia:** Baseline information of the participants; n = 32 of each group.

Variable	Value	p-value
Patients; count in each group	32	
*Age; Mean±SD (range) years*		
Study group	43.84±6.7 (34-60)	0.29
Control	41.12±4.8 (32-49)	
*Gender; count (%)*		
Male	52 (81.3)	
Female	12 (18.7)	
*Stone laterization; count (%)*		
Center	36 (62.5)	
Right	28 (37.5)	
*Stone size; Mean±SD (range) mm*		
Study group	5.47±1.0 (3.4-6.8)	0.0001
Control	4.50±0.6 (3.2-5.4)	

Predictors for stone expulsion were studied after noticing poor stone expulsion rate i.e. 81.2% (n = 26) in control group (Table-Ib). Comparatively lower values of stone expulsion rate were seen in patients: aged ≤40 years (77.3%, n = 17), males (75%, n = 18), having stone in left ureter (70%, n = 14), or stone size of >5mm (50%, n = 1). However, the rate showed insignificant association with any of the variables except stone laterization (*p*>05). The therapy was approximately 2-fold less effective in expulsion of stone from left ureter (OR=1.86; 95% CI: 1.30-2.65) than right ureters (*p* = 0.04).

**Table-Ib:** Risk estimates in participants of control group (n = 32).

Variable	Stone expulsion; % (n)	Odd ratio; 95% CI	p-value
*Age (in years)*			
≤40	77.3 (17)	2.65; 0.27-26.25	0.37
>40	90 (9)		
*Gender*			
Male	75 (18)	1.44; 1.12-1.87	0.15
Female	100 (8)		
*Stone laterization*			
Center	70 (14)	1.86; 1.30-2.65	0.04
Right	100 (12)		
*Stone size (in mm)*			
≤5	83.3 (25)	0.20; 0.01-3.76	0.35
> 5	50 (1)		

Data presented in Table-II show cent percent stone expulsion (n = 32) against therapy using tamsulosin with phloroglucinol for stone expulsion. However, risk estimates revealed that a patient treated with mono therapy had lesser chances of stone expulsion (RR: 0.812; 95% CI: 0.688-0.960) compared to that of combination therapy (*p* = 0.02).

**Table-II:** Therapy vs. stone expulsion rate; and risk estimates.

Characteristic	Value
*Stone expulsion; % (n)*	
Combination therapy	100 (32)
Mono therapy	81.2 (26*)
Relative risk estimates	RR = 0.812; 95% CI: 0.688 – 0.960; p = .02

* In18.8% (n = 6) subjects, the stone was removed by extracorporeal lithotripsy

Data in Table-III depicts comparative accounts of medication time in phloroglucinol-added and –free therapies. Combination therapy had advantage on mono therapy as it expelled the distal ureteral stone in significantly lower mean time, 10.34 (*SD* = 3.5; range 3-15) days after start of medication (*p* = .0001). The therapy also showed efficacy against both, small (≤5) and large-sized stones (>5 mm) by removing all such stones i.e. 6 (18.8%) and 26 (81.2%) within 7 and 15 days of treatment, respectively.

**Table-III:** Medication days vs. type of medical expulsion therapy.

Variable	Value	
Medication time; Mean+/-SD (range) days		
Combination therapy	10.34 ±3.5 (3-15)	
Mono therapy	17.69 ±2.8 (9-21)	(p = .0001)
Medication time; Range (n, %) days		
Combination therapy (≤5 mm-sized stone)	1-7 (6, 18.8)	
Mono therapy…	8-14 (4, 12.5), 15-21 (21, 65.6)	
Combination therapy (>5 mm-sized stone)	1-7 (1, 3.1), 8-14 (24, 75.0), 15-21 (1*, 3.1)	
Mono therapy	15-21 (1**, 3.1)	

* 15^th^and

** 20^th^day of medication

Colic episode needed couples use of analgesic (50mg diclophenac Na). The patients of study group reported significantly lesser numbers of pain incidence (*M* = 0.19, *SD* =0.4) as shown in Table-IV (*p* =.03). So, a remarkable difference in mean consumption of analgesic (12.50 vs. 29.69 mg) was noticed between study and control groups to relieve pain (*p* = .02). Only one patient of study group had to visit emergency room without being hospitalized. However, one subject from control had to stay at hospital for certain complications.

**Table-IV:** Colic episode/analgesic vs. type of therapy.

Variable	Value	p-value
*Colic episode; Mean+/-SD (range) count*		.
Combination therapy	0.19±0.4 (0-1)	03
Mono therapy	0.52±0.6 (0-2)	
*Analgesic; Mean+/-SD (range) mg*		
Combination therapy	12.50±22.0 (0-50)	.02
Mono therapy	29.69±30.8 (0-100)	
*Visit of emergency room; count*		
Combination therapy	1	
Mono therapy	4	
*Hospitalization; count*		
Combination therapy	Nil	
Mono therapy	1	

**Fig.2:**
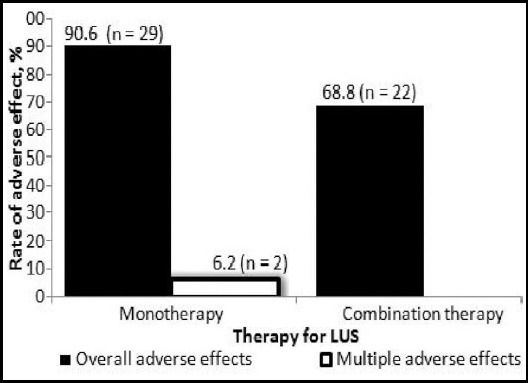
Rate of adverse effect against therapies for LUS (n = 32).

Incidence of adverse effects against both therapies is shownin Fig.2. In patients with phloroglucinol-added therapy, the rate 68.8% (n = 22) was comparatively lower. Moreover, asthenia was found as the most prevalent adverse effect of present investigation. Other effects included headache, orthostatic hypotension, palpitation, nausea/vomiting, and gastro intestinal disorders. Additionally, the phloroglucinol-added medication showed its advantage on having no multiple adverse signs.

## DISCUSSION

The LUS (lower ureteral stone)-mediated colic pain is associated with a sense of unknown fear and subsequently declination towards any activity. Radom group allotment rules out any risk of bias^11^ in subject sampling. Similarly, leaving the study (within the session) is the consequence of self-perceived poor prognosis,^12^ outcome of adverse effects or any other drive e.g. economic constrains.

Mean age of participants in present work (early 40’s) looks quite different from that of late 40’s, mid 30’s, or 20’s.^8^,^10^,^12^ The deviation may have differences in life style including dietary habits, and climate. Females are usually less prone to the LUS.^13^,^14^ Our results i.e. 12 out of 64 (i.e. 18.8%) substantiate this general concept. The mark difference can be interpreted with respect to sex-linked endocrinology. Comparatively higher rate of stones in left ureter is just a chance otherwise 1:1 ratio is expected.^15^ Just like in a published work^16^, the mean value of stone (about 5 mm) indicates the commonness of this size in the patients of LUS.

Output of insignificant association between stone expulsion rate and baseline information of the control group (tamsulosin alone) reveals equal impacts of the MET on both the categories of each characteristic. Almost similar findings has been documented by authors^15^, working on rate of ureteroscopy for the LUS. Failure to expel some stones from left ureter is surprising and can be traced back to some risk factors such as uneven surface of the stone and/or irregularity in medication.

The 100% stone expulsion rate against phloroglucinol-added tamsulosin therapy of present investigation is higher than previously reported for sole phloroglucinol (64%),^6^,^22^ tamsulosin,^21^-^24^ tadalafil-added tamsulosin,^8^,^17^ or tolterodine-added tamsulosin.^20^ The better efficacy of tamsulosin plus phloroglucinol medication is a good example of synchronization impact of the drugs towards the desired purpose i.e. stone expulsion. Luckily, combination therapy discourages drug tolerance in the patients.

The MET is the choice of people agreed to waiting management for the LUS. Our combination therapy expelled the stones in lesser mean time (in days) than already reported 8,17 using other set of medicines e.g. tadalafil-added tamsulosin. Almost equal affectivity of our combination modality for both, small and large-sized stones advocate its future prospective in medicinal world.

Decline in frequency of the colic episodes in combination therapy marks the anti-spasmodic role of added phloroglucinol.^6^ Decline in number of patients with the colic episode(s) in the combination (n=6; 18.8% compared to monotherapy n=14; 43.8%) is steeper than parecoxib (n =17; 14.3%) and parecoxib plus phloroglucinol (n=7; 6.1%) of a published data.^18^ It appears that phloroglucinol becomes more effective when it is in partnership with tamsulosin. Decrease in demand of analgesic in combination therapy makes the therapy cost effective.

Comprehensive reporting of the adverse effects is required along with medicine to balance its overemphasized benefits.^19^ The combination therapy of present work shows advantage over matched tamsulosin alone in term of rate of the effects.

## CONCLUSION

Combination therapy has advantage over monotherapy in term of stone expulsion rate, medication time, colic episodes, use of analgesic drugs, and adverse side effects. The findings will guide the researchers to retest the applicability of the modality at higher levels before general application.

### Authors’ Contribution

**MNS** conceived, designed, collected data and wrote manuscript.

**MH** applied statistical analyses; edited, reviewed and approved the manuscript.
